# The use of a large language model to create plain language summaries of evidence reviews in healthcare: A feasibility study

**DOI:** 10.1002/cesm.12041

**Published:** 2024-02-04

**Authors:** Colleen Ovelman, Shannon Kugley, Gerald Gartlehner, Meera Viswanathan

**Affiliations:** ^1^ Center for Public Health Methods RTI International Research Triangle Park North Carolina USA; ^2^ Central Editorial Service Evidence Production & Methods Directorate, Cochrane London UK; ^3^ Department for Evidence‐based Medicine and Evaluation University of Krems Krems Austria

## Abstract

**Introduction:**

Plain language summaries (PLSs) make complex healthcare evidence accessible to patients and the public. Large language models (LLMs) may assist in generating accurate, readable PLSs. This study explored using the LLM Claude 2 to create PLSs of evidence reviews from the Agency for Healthcare Research and Quality (AHRQ) Effective Health Care Program.

**Methods:**

We selected 10 evidence reviews published from 2021 to 2023, representing a range of methods and topics. We iteratively developed a prompt to guide Claude 2 in creating PLSs which included specifications for plain language, reading level, length, organizational structure, active voice, and inclusive language. PLSs were assessed for adherence to prompt specifications, comprehensiveness, accuracy, readability, and cultural sensitivity.

**Results:**

All PLSs met the word count. We judged one PLS as fully comprehensive; seven mostly comprehensive. We judged two PLSs as fully capturing the PICO elements; five with minor PICO errors. We judged three PLSs as accurately reporting the results; and four with minor result errors. We judged three PLSs as having major result errors for incorrectly reporting total participants. Five PLSs met the target 6th to 8th grade reading level. Passive voice use averaged 16%. All PLSs used inclusive language.

**Conclusions:**

LLMs show promise for assisting in PLS creation but likely require human input to ensure accuracy, comprehensiveness, and the appropriate nuances of interpretation. Iterative prompt refinement may improve results and address the needs of specific reviews and audiences. As text‐only summaries, the AI‐generated PLSs could not meet all consumer communication criteria, such as textual design and visual representations. Further testing should include consumer reviewers and explore how to best leverage LLM support in drafting PLS text for complex evidence reviews.

## 1 BACKGROUND

Evidence reviews, including systematic reviews, of healthcare research comprehensively collect and analyze information across studies to support best practices and policy for a wide range of topics and questions [1, 2]. They may include data from a few to hundreds of studies and address specific questions including efficacy, safety, and prognosis, among others. These reviews are frequently lengthy and complex, containing scientific terms, challenging concepts, and sophisticated analyses.

Funders, publishers, and producers of systematic reviews of healthcare research support and often require the use of a plain language summary (PLS) to communicate complex information to the public. Government agencies and funders, such as the US National Institutes of Health (NIH) [3] and the UK National Institute of Health and Care Research (NIHR) [4], explicitly advocate for plain language materials that use common, everyday words to relay information clearly and concisely, as a way to ensure that health research is accessible and clear for all readers. The US Agency for Healthcare Research and Quality (AHRQ) in collaboration with its Evidence‐based Practice Centers (EPCs), supports the production of evidence reviews including comparative effectiveness reviews (CER) [5]. In compliance with the 2010 Plain Writing Act, AHRQ requires the use of plain language when the agency creates content for the public [6]. Likewise, Cochrane, which produces the leading database for healthcare‐related systematic reviews, requires PLSs for all reviews published in the Cochrane Library [7].

Key components of PLSs include the use of the active voice, logical organization, short sentences and paragraphs, avoidance of jargon and technical terms, and the definition of such terms, if they must be used [4, 7, 8]. In general, PLSs should be written at the reading level of their intended audience. While specific reading level guidance is sometimes given [4], most current guidance, such as that developed by AHRQ and Cochrane [7], note that reading level varies by the context of the audience so should be tailored to the context, and that existing formulas used to assess reading levels are best used as tools to diagnose overly complex text [9]. Other expectations of plain language materials created for healthcare communication include layout, the use of tables or graphics, and specific typography [8].

Even with existing guidance, crafting a PLS of reports that include scientific evidence and terminology may be challenging for researchers [10]. Analyses of PLSs published in Cochrane reviews found variability in their quality, readability, accuracy, and adherence to plain language recommendations [11] (i.e., Plain Language Expectations for Authors of Cochrane Summaries [PLEACS], superseded by newer Cochrane guidance [12]).

Recent advances in artificial intelligence, particularly large language models (LLM), offer an opportunity to assist authors in the creation of PLSs. While a LLM cannot meet all of the criteria expected for consumer communications [8]—use of images, graphics, specific typography, for example—it may be able to create the text portions of such communications or meet the needs of PLSs for journal publications, such as Cochrane [12].

There are several LLMs publicly available, including ChatGPT [13], Bard [14], and Claude [15]. Claude 2, developed by Anthropic (https://claude.ai/chats) and launched in July 2023, includes enhanced content generation and coding skills, and supports the direct upload of Portable Document Formats (PDFs) [16]. Claude 2 training data was updated in 2022 and early 2023 [17].

## 2 OBJECTIVE

To explore the feasibility of using the LLM, Claude 2, to develop PLSs of evidence reviews that are appropriate for patients and consumers.

## 3 METHODS

We selected a purposive sample of 10 AHRQ EPC reviews published from 2021 to present [18, 19, 20, 21, 22, 23, 24, 25, 26, 27]. The sample included systematic reviews using a variety of synthesis methods, of varying complexity, and presenting both quantitative and qualitative results.

We created a PDF of the executive summary from each report. We created a prompt including the parameters of a PLS based upon AHRQ's and Cochrane's plain language requirements [6, 12]. The prompt included specifications for plain language, reading level, length, organizational structure, active voice, and inclusive language, following the American Association of Psychologists' inclusive language guidance [28]. We tested the prompt on a sample executive summary and made minor adjustments before a final version was created. See Supporting Information S1: Appendix 1 for the full prompt.

As Claude consistently created PLSs which were very brief (550 words or less), we used a second prompt (Supporting Information S1: Appendix 2) requesting more information to be included in the summary. If Claude had not provided the number of included studies and patients in the first version of the summary, we also asked it to include those as part of the second prompt.

### 3.1 Data collection and analysis

We created a data collection form to assess the AI‐generated summaries based on comprehensiveness, accuracy, readability, and cultural sensitivity of the language used (Table 1). We had planned to also use the Centers for Disease Control and Preventions' (CDC) Clear Communication Index as a tool, but it did not adequately assess the summaries, as it measures use of graphics, tables and other presentation styles not applicable to a text‐based summary [30].

**Table 1:** Data collection form.

Feature	Description	Tool(s)
Length	Within the word count specified in the prompt: ≤850 words	MS Word: word count feature
Organization	Follows the organization outline in the prompt	No tools used/available
Accuracy: PICO	Includes correctly articulated dimensions of PICO (yes [0 errors], mostly yes [1–3 minor errors], no (more than 3 minor errors or 1 major error)	No tools used/available
Accuracy: Results	Results reported are accurate (yes [0 errors], mostly yes [1–3 minor errors], no [more than 3 minor errors or 1 major error])	No tools used/available
Comprehensiveness	Comparison of PLS with the ES to check it has captured all key evidence points (yes [0 missing], mostly yes [1–3 missing], no [more than 3 missing])	No tools used/available
Readability: grade level	Measured as a binary outcoming using 2 different scales as either within or not within 6‐8th grade reading level; used an additional formula, Flesch‐Kincaid readability score, and measured as a binary yes/no for scoring between 60‐70 (considered Plain English) [9, 29]	Used 3 validated formulae: Simple Measure of Gobbledygook (SMOG), Flesch‐Kincaid Grade Level, and Flesch‐Kincaid Read Ease formulae which can be applied via this tool: http://www.readabilityformulas.com/free-readability-formula-tests.php)
Readability: active versus passive voice	Passive voice percentage	MS Word Editor
Definition of complex medical terms	Medical terms are defined or described in a way that is understandable to nonclinical/technical readers at first usage	No tools used/available
Culturally appropriate	Editor settings set for all measures of inclusivity and sensitive references	MS Word Editor: inclusivity and sensitive geopolitical references editor

*Note*: One reviewer assessed each PLS, and a second reviewer conducted a quality check. Disagreements were resolved via discussion. We include a description of the comprehensiveness and accuracy findings in Supporting Information S1: Appendix 5.

For comprehensiveness, we compared the AI‐generated PLS to the executive summary to check if it captured all key evidence points and rated the summaries with yes, mostly yes (missing one to three items) or no (for those missing more than three items). We measured accuracy in two domains: the first for reporting the PICO (population, intervention, comparator, outcomes) elements of the evidence review, and the second for reporting the results of the review. For accuracy of the PICO elements and the results, we also used the following rating system: yes, mostly yes (one to three minor errors), or no (more than three minor errors or one major error). For accuracy of PICO and results reporting, we considered minor errors as those which were not completely correct but did not threaten overall understanding. The following were considered major errors: any error in numerical results data; any hallucination (a number reported in the AI‐generated summary which did not appear in the executive summary); and any summary statement, PICO element, or reporting of qualitative evidence that was pointedly misleading. Further description of each assessment category is available in Table 1. One author independently assessed each summary and completed the data collection form. A second author then checked the assessments. We resolved disagreements via discussion.

## 4 RESULTS

### 4.1 Sample

The sample included 10 executive summaries from recently published EPC reviews (Supporting Information S1: Appendix 3) [18, 19, 20, 21, 22, 23, 24, 25, 26, 27]. All were systematic reviews, utilizing a variety of systematic review methods. One of the included EPC reviews was an update [18]. Five were published in 2021, four in 2022 and one in 2023. The reviews were authored by seven EPCs (two by ECRI, two by Johns Hopkins, two by RTI, and one each from Minnesota, Pacific NW, and RAND).

### 4.2 AI‐generated PLSs

All 10 of the AI‐generated PLSs were within the word limit of at or below 850 words, even after applying the second prompt to expand the summary and increase the word count.

#### 4.2.1 Comprehensiveness

In terms of comprehensiveness, we judged one PLS as capturing all key evidence points included in the ES; seven as being mostly comprehensive (with one to three missing evidence points); and one as missing more than three evidence points (Table 2). Important details were sometimes missed. In a review of vaccine safety [18], the AI‐generated PLS lumped together findings for “vaccines routinely given to children” rather than identifying the specific vaccines.

**Table 2:** Results: Comprehensiveness and accuracy.

Comprehensiveness	Yes (0 missing)	Mostly yes (1–3 missing)	No (more than 3 missing)
Captures all key evidence points included in the ES	1	7	2
Accuracy	Yes (0 errors)	Mostly yes (1‐3 minor errors)	No (more than 3 minor errors)
Dimensions of PICO described correctly	2	5	3
Results described correctly	3	4	3

Abbreviations: ES, executive summary; PICO, population, intervention, control, outcomes.

#### 4.2.2 Accuracy

Two PLSs accurately captured all elements of the PICO (patients, interventions, comparators, outcomes) presented in the ES (Table 2). Five PLSs included 1–3 minor errors in this domain, and three PLSs included either more than three minor errors or one major error.

Three PLSs accurately reported the results (Table 2). We judged four as having 1–3 minor errors in results reporting, and three as having three or more minor errors or one major error in results reporting. Of note, all three in this final category were judged as having a major error because they misreported (potential “hallucination”) the number of included participants. In these instances, the authors of the executive summaries did not include the total number of included participants in the original summaries.

One EPC review was an updated review [18]. The AI‐generated PLS reported the evidence in total rather than reporting separately on what the updated review added to the previous version of the review. For this, we judged the PLS as falling into the minor error category.

#### 4.2.3 Readability and style

All PLSs followed the organizational structure in the prompt, except for two which initially followed the organizational structure in the prompt, but added an additional summary at the end of the PLS when the second prompt was applied (Table 3).

**Table 3:** Results: Readability and style.

Length	Yes	No
≤850 words	10	0
Organizational structure	Yes	No
Followed the prompt	8	2
Readability: 6–8th grade reading level	Yes	No
Average results from three validated scales^a^	5	5
Readability: active versus passive voice	Average	Range
Use of passive voice^b^	16%	7%–28%
Readability: complex medical terminology	Yes (all terms defined at 1st usage)	Mostly yes (all but 1–3 terms defined at 1st usage)
Defined at first usage	7	3
Style: inclusive language	Yes	No
Use of culturally appropriate language^b^	10	0

^a^ Validated formulae: Simple Measure of Gobbledygook (SMOG), Flesch‐Kincaid Grade Level, and Flesch‐Kincaid Read Ease.

^b^ Measured with the MS Word Editor tool.

For further assessment of readability, we used three, validated reading level scales (Simple Measure of Gobbledygook (SMOG), Flesch‐Kincaid Grade Level, and Flesch‐Kincaid Read Ease formulae). In combining the average results from all three scales, half (5 of the 10) met the 6th to 8th grade reading level criteria we included in the prompt (Table 3). AHRQ notes that such formulae can highlight signals in text readability but are not appropriate for overall assessments of comprehension [9].

Use of passive voice within the AI‐generated PLSs ranged from 7% to 28% (avg: 16%; Table 3). We assessed seven PLSs as having defined all complex medical terms at first usage, while three were judged as defining most (with the exception of 1–3 terms) at first usage. All PLSs met the criteria for using inclusive language.

Qualitatively, we noted that the style across AI‐generated PLSs was inconsistent. When using the same prompt for a specific ES, or using additional prompts, we noted that the sentence constructions sometimes shifted in the text that Claude produced.

## 5 CONCLUSIONS

We examined the performance of AI‐generated PLSs of 10 AHRQ EPC systematic reviews covering a range of different health topics and published in the last 2 years. Our assessment suggests that large language models, such as Claude, have the potential to assist authors in creating PLSs for complex evidence reviews. While a standard prompt could be helpful for this, the prompt will need engineering to meet the specific needs of the audience or to adequately capture the array of information in complex reviews. For example, if the review is an update, the AI prompt would need to be engineered to capture the information authors would like to convey. For number of patients, the prompt likely needs an additional statement that tells the LLM not to predict a number if it is not reported explicitly in the text.

A solely text‐based PLS such as the ones created for this project, do not meet the criteria for consumer communication as outlined by organizations such as the CDC, which suggest that healthcare communication include images, visual representations, and textual considerations such as font size, layout, and the use of bolder headers. However, a PLS generated with a LLM could be used to create a first draft of the text portions of such a consumer product.

Any AI‐generated material will likely need a human reviewer to confirm accuracy, check for comprehensiveness, and add appropriate nuanced interpretation. Further testing is needed to understand how to best leverage LLMs to support the development of PLSs of complex evidence reviews. Such testing should include consumer reviewers and could expand the current study to include a blinded comparison of AI‐generated versus human‐generated PLSs.

### 5.1 Limitations

As a feasibility study, there are limitations to this project. During this initial testing, we did not include consumers to assess the AI‐generated summaries. As the target audience of PLSs, future studies should include consumer reviewers.

Next, we chose to include just the executive summaries instead of the PDF of the full report when prompting Claude to create the PLSs. Claude 2 has the capability to upload a full report PDF, and the output may vary based on the use of more extensive source material.

Some of the assessments we included were subjective (e.g., accuracy and comprehensiveness). Repeat assessments conducted by other assessors may differ, especially if conducted by individuals with content or subject matter expertise matching the review topic. For evaluating major and minor errors in comprehensiveness and accuracy, one author conducted assessments and a second author checked these assessments. Dual independent assessments may have led to different performance results. For determining the use of culturally sensitive and inclusive language, we relied upon a function within Microsoft Word to evaluate this and did not perform our own independent evaluation.

Additionally, we included a limited set of evidence reviews. A broader range of topics and review types (e.g., rapid reviews, health technology assessments, etc.) may influence the findings. Finally, we did not measure comprehension per se. While the readability formulae we used are validated, they have limitations. Achieving a 6th to 8th grade reading level for complex health topics is challenging and when achieved, does not necessarily ensure that the material is comprehensible. To fully understand the quality and usefulness of AI‐generated PLSs, engagement with consumers who can assess the summaries is necessary.

Lastly, LLMs, their features, and underlying models are evolving. The replicability of the LLM output is unknown. Future investigations should examine the performance using the same prompts and materials over time and using different LLMs.

## AUTHOR CONTRIBUTIONS

**Colleen Ovelman**: Conceptualization; data curation; formal analysis; investigation; methodology; writing—original draft. **Shannon Kugley**: Conceptualization; data curation; formal analysis; investigation; methodology; writing—review and editing. **Gerald Gartlehner**: Conceptualization; investigation; methodology; resources; supervision; writing—review and editing. **Meera Viswanathan**: Conceptualization; funding acquisition; investigation; methodology; resources; supervision; writing—review and editing.

## CONFLICT OF INTEREST STATEMENT

The authors declare no conflict of interest.

## PEER REVIEW

The peer review history for this article is available at https://www.webofscience.com/api/gateway/wos/peer-review/10.1002/cesm.12041.

## Supporting information

Supporting information.

## Data Availability

Data are available from the corresponding author upon request.
