# Supplementary material for: The use of a large language model to create plain language summaries of evidence reviews in healthcare: A feasibility study
Source: Cochrane Evid Synth Methods. 2024 Feb 4;2(2):e12041. doi: 10.1002/cesm.12041 (PMC11795904; doi:10.1002/cesm.12041)
Supplement: Supplementary file 1 — Supporting information. [file CESM-2-e12041-s001.docx]

# Appendix 1: Prompt for the LLM

The attached document is an abstract of a comparative effectiveness review entitled, “XXX.”

Act as a science researcher who wants to explain complex medical information to the public. Please create a plain language summary of this abstract that includes all of the same information but is written at a sixth to eighth grade reading level. Keep the summary close to but not exceeding 850 words. Write in the active voice. Use the first-person plural pronoun “we” to describe what the researchers have done. Explain common medical words and complex medical terms when they are first used. Use inclusive language, as outlined in the American Psychological Association’s Inclusive Language Guidelines, such as person-first or identity-first language as is appropriate for the community or person being discussed.

Include a plain language title.

Organize the summary into the following headings:

• Key messages

• A heading about the health condition of the included patients, for example, What is epilepsy?

• A heading about the treatment being studied, for example, How is epilepsy treated?

• What did we want to find out?

• What did we do?

• What did we find?

• What are the limitations of the evidence?

• How up to date is this evidence?

# Appendix 2: Second Prompt for the LLM, as needed

1. This is good, but this summary is only X words. Can you add more information from the attached abstract to this plain language summary following all of the criteria in the first prompt while getting the plain language summary closer to 850 words but ensuring that it does not exceed 850 words?
2. This is good, but this summary is only X words. Can you add more information from the attached abstract to this plain language summary following all of the criteria in the first prompt while getting the plain language summary closer to 850 words but ensuring that it does not exceed 850 words? 
   Please include in the summary the total number of included studies and patients.

# Appendix 3: Systematic Reviews Included for PLS

| **Authoring EPC** | **Internet citation** | **Year published** | **Product type** | **CER number** | **Topic** | **URL** |
| --- | --- | --- | --- | --- | --- | --- |
| ECRI | Systematic Review: Malnutrition in Hospitalized Adults. Content last reviewed April 2022. Effective Health Care Program, Agency for Healthcare Research and Quality, Rockville, MD. | 2021 | systematic review | CER 249 | malnutrition | https://effectivehealthcare.ahrq.gov/products/malnutrition-hospitalized-adults/research |
| ECRI | Systematic Review: Management of Infantile Epilepsies. Content last reviewed March 2023. Effective Health Care Program, Agency for Healthcare Research and Quality, Rockville, MD. | 2022 | systematic review | CER 252 | infant epilepsy | https://effectivehealthcare.ahrq.gov/products/management-infantile-epilepsy/research |
| JHU | Systematic Review: Diagnostic Errors in the Emergency Department: A Systematic Review. Content last reviewed February 2023. Effective Health Care Program, Agency for Healthcare Research and Quality, Rockville, MD | 2022 | systematic review | CER 258 | diagnostic errors | https://effectivehealthcare.ahrq.gov/products/diagnostic-errors-emergency/research |
| JHU | Systematic Review: Integrating Palliative Care in Ambulatory Care of Noncancer Serious Chronic Illness: A Mixed-Methods Review. Content last reviewed March 2022. Effective Health Care Program, Agency for Healthcare Research and Quality, Rockville, MD. | 2022 | systematic review | CER 237 | palliative care | https://effectivehealthcare.ahrq.gov/products/palliative-care-integration/research |
| Mayo | Systematic Review: Partial Breast Irradiation for Breast Cancer. Content last reviewed May 2023. Effective Health Care Program, Agency for Healthcare Research and Quality, Rockville, MD. | 2023 | systematic review | CER 259 | irradiation for early-stage breast cancer | https://effectivehealthcare.ahrq.gov/products/partial-breast-irradiation/research |
| Minnesota | Transitions of Care From Pediatric to Adult Services for Children With Special Healthcare Needs. Content last reviewed October 2022. Effective Health Care Program, Agency for Healthcare Research and Quality, Rockville, MD. | 2022 | systematic review | CER 255 | pediatric to adult transition of care | https://effectivehealthcare.ahrq.gov/products/transitions-care-pediatric-adult/research |
| Pacific NW | Systematic Review: Prehospital Airway Management. Content last reviewed August 2021. Effective Health Care Program, Agency for Healthcare Research and Quality, Rockville, MD. | 2021 | systematic review | CER 243 | airway management | https://effectivehealthcare.ahrq.gov/products/prehospital-airway-management/research |
| RAND | Systematic Review: Safety of Vaccines Used for Routine Immunization in the United States: An Update. Content last reviewed January 2022. Effective Health Care Program, Agency for Healthcare Research and Quality, Rockville, MD. | 2021 | systematic review (update) | CER 244 | vaccination safety | https://effectivehealthcare.ahrq.gov/products/safety-vaccines/research |
| RTI | Systematic Review: Management of High-Need, High-Cost Patients: A “Best Fit” Framework Synthesis, Realist Review, and Systematic Review. Content last reviewed December 2022. Effective Health Care Program, Agency for Healthcare Research and Quality, Rockville, MD | 2021 | systematic review | CER 246 | management of HCHN patients | https://effectivehealthcare.ahrq.gov/products/high-utilizers-health-care/research |
| RTI | Systematic Review: Maternal, Fetal, and Child Outcomes of Mental Health Treatments in Women: A Systematic Review of Perinatal Pharmacologic Interventions. Content last reviewed March 2022. Effective Health Care Program, Agency for Healthcare Research and Quality, Rockville, MD. | 2021 | systematic review | CER 236 | pharmacologic interventions for mental health | https://effectivehealthcare.ahrq.gov/products/mental-health-pregnancy/research |

# Appendix 4 : AI-generated PLS Assessment Results

| **CER Number** | **Review Title** | **Year** | **Word count** | **Organization** | **Comprehensiveness** | **Accuracy (PICO)** | **Accuracy (results)** | **SMOG 6-8** | **Flesch-Kincaid 6-8** | **Flesch-Kincaid Read Ease 60-70** | **Passive voice, %** | **Defined terms** | **Culturally appropriate language** |
| --- | --- | --- | --- | --- | --- | --- | --- | --- | --- | --- | --- | --- | --- |
| 255 | Transitions of Care From Pediatric to Adult Services for Children With Special Healthcare Needs | 2022 | ≤ 850 | Yes | Mostly yes (1-3 missing) | No (more than 3 minor errors; at least one major error) | No (more than 3 minor errors; at least one major error) | Yes | Yes | Yes | 7.1 | Yes, all terms defined at first use | Yes |
| 236 | Maternal, Fetal, and Child Outcomes of Mental Health Treatments in Women: A Systematic Review of Perinatal Pharmacologic Interventions | 2021 | ≤ 850 | Yes | Mostly yes (1-3 missing) | Yes | Mostly yes (1-3 minor errors) | No | No | No | 9.7 | Most terms defined at first use (1-3 are not) | Yes |
| 246 | Management of High-Need, High-Cost Patients: A "Best Fit" Framework Synthesis, Realist Review, and Systematic Review | 2021 | ≤ 850 | Yes | No (more than 3 missing) | Yes | Yes | Yes | Yes | Yes | 9.8 | Yes, all terms defined at first use | Yes |
| 252 | Management of Infantile Epilepsies | 2022 | ≤ 850 | Yes | Yes | No (more than 3 minor errors; at least one major error) | Mostly yes (1-3 minor errors) | Yes | No | No | 12.9 | Yes, all terms defined at first use | Yes |
| 249 | Malnutrition in Hospitalized Adults: A Systematic Review | 2021 | ≤ 850 | Yes | Mostly yes (1-3 missing) | Mostly yes (1-3 minor errors) | No (more than 3 minor errors; at least one major error) | Yes | Yes | No | 15 | Yes, all terms defined at first use | Yes |
| 244 | Safety of Vaccines Used for Routine Immunization in the United States: An Update | 2021 | ≤ 850 | No | No (more than 3 missing) | Mostly yes (1-3 minor errors) | Mostly yes (1-3 minor errors) | No | No | No | 16 | Yes, all terms defined at first use | Yes |
| 237 | Integrating Palliative Care in Ambulatory Care of Noncancer Serious Chronic Illness: A Mixed-Methods Review | 2022 | ≤ 850 | Yes | Mostly yes (1-3 missing) | Mostly yes (1-3 minor errors) | Yes | No | No | No | 17.2 | Most terms defined at first use (1-3 are not) | Yes |
| 259 | Partial Breast Irradiation for Breast Cancer | 2023 | ≤ 850 | Yes | Mostly yes (1-3 missing) | Mostly yes (1-3 minor errors) | Mostly yes (1-3 minor errors) | Yes | Yes | Yes | 24.3 | Yes, all terms defined at first use | Yes |
| 243 | Prehospital Airway Management: A Systematic Review | 2021 | ≤ 850 | No | Mostly yes (1-3 missing) | Mostly yes (1-3 minor errors) | Yes | No | No | No | 26.8 | Yes, all terms defined at first use | Yes |
| 258 | Diagnostic Errors in the Emergency Department: A Systematic Review | 2022 | ≤ 850 | Yes | Mostly yes (1-3 missing) | No (more than 3 minor errors; at least one major error) | No (more than 3 minor errors; at least one major error) | Yes | Yes | No | 28 | Most terms defined at first use (1-3 are not) | Yes |

# Appendix 5. Description of comprehensiveness and accuracy findings

| **CER number** | **Title** | **Description of missed and inaccurate information in the Claude 2 generated PLSs** |
| --- | --- | --- |
| CER 236 | Maternal, Fetal, and Child Outcomes of Mental Health Treatments in Women: A Systematic Review of Perinatal Pharmacologic Interventions | PLS did not indicated that medication risks were specific to the first trimester of pregnancy |
| CER 237 | Integrating Palliative Care in Ambulatory Care of Noncancer Serious Chronic Illness: A Mixed-Methods Review | PLS indicated "serious chronic illness" but does not indicate “excluding cancer” (the review omitted cancer because another review was recently completed); PLS did not include stakeholder feedback which was a key source of information for the review. |
| CER 243 | Prehospital Airway Management | PLS indicated that the review "directly compared at least two of these airway management methods" but the review also compared different approaches or variations of one type of airway management method, such as video and direct laryngoscopy; PLS description of methods missed "within ETI (RSI comparisons)"; PLS findings missed: “When SGA was compared with ETI, outcomes measured by the CPC favored ETI in adult patients with cardiac arrest”; PLS included a sentence in the currency of evidence section that should have been included as a key message or in the results section: "The results highlight that each method may have a role depending on the patient and situation. More research can help determine guidelines for emergency medical staff on the best approaches to use." |
| CER 244 | Safety of Vaccines Used for Routine Immunization in the United States: An Update | This is a topic for which the ES tables of individual vaccines and the symbols used were important and not captured adequately in the AI-generated text version; PLS did not make clear that the summary was an update to a prior report; results did not include vaccines with insufficient evidence; PLS did not list the vaccines associated with side rare side effects (i.e., allergic reaction and low platelet count); PLS included a statement in the currency of evidence section that should have been in the key messages or results |
| CER 246 | Management of High-Need, High-Cost Patients: A “Best Fit” Framework Synthesis, Realist Review, and Systematic Review | PLS oversimplified what was done (no mention of best-fit framework synthesis and realist review); PLS missed the following key findings: “Both patients and care providers require support and practical resources to foster an effective relationship” and description of the three program theories that emerged from the review of evidence” |
| CER 249 | Malnutrition in Hospitalized Adults | PLS included an inaccurate description of key message: "Malnutrition in the hospital links to higher death rates, longer hospital stays, and more complications. Quickly screening all patients to find malnutrition risk is important." as it missed versus well-nourished and ICU; unable to confirm the number of participants that the PLS listed: "over 5000"; PLS referred to "new studies" |
| CER 252 | Management of Infantile Epilepsies | PLS included redundant information in description of epilepsy: "Having uncontrolled seizures as a baby may affect development, behavior, thinking skills, and quality of life. But treatments also have risks. Doctors and parents must balance controlling seizures with avoiding treatment harms. Uncontrolled seizures have serious health risks for babies. But treatments like medicines can also harm thinking skills and development."; PLS listed medications not listed in report and omitted medications listed in the report (i.e., phenytoin and stiripentol): PLS described a diet treatment that was not described in report and the description was inaccurate: "The ketogenic diet is very high in fat and low in carbs and protein" as ketogenic diet is high in both protein and fat |
| CER 255 | Transitions of Care from Pediatric to Adult Services for Children With Special Healthcare Needs | PLS used an inaccurate title that did not mention CHSCNs: "Helping Kids Transition to Adult Healthcare"; PLS missed KQ3 in description of "What did we want to find out?"; PLS missed two key findings: implementation findings and variation in training and interventions to prepare CSHCNs for transition |
| CER 258 | Diagnostic Errors in the Emergency Department: A Systematic Review | PLS used an inaccurate title as the review examined errors could be due to system factors as well as care team or provider and reasons for the errors: "Common Types of Mistakes Doctors Make in Emergency Departments and Their Causes "; unable to confirm the number patients listed by the PLS: "We found 279 relevant studies involving over 1.7 million patients."; PLS included an inaccurate finding: "Stroke, heart attack, and spinal injuries accounted for 39% of serious misdiagnoses." 5 conditions account for 39 percent of misdiagnoses; PLS included a statement that did not represent the nuanced implications and conclusions in the report: "Our findings suggest diagnostic errors cause substantial preventable harm in emergency departments." |
| CER 259 | Partial Breast Irradiation for Breast Cancer | PLS included information not in the report: "is typically given 5 days a week for 3-6 weeks" and "PBI allows completing treatment in just 1-2 weeks"; PLS missed "financial toxicity" as an outcome of interest; PLS stated an incorrect number of studies: "We reviewed results from 23 high quality studies comparing PBI to WBI" should be 13 RCTs; PLS did not include the following information in the "What did we find" section: financial results, results from different types of PBI, and results for intraoperative radiotherapy |
